# Linking gut microbiota with the human diseases

**DOI:** 10.6026/97320630016196

**Published:** 2020-02-29

**Authors:** Udaya Kumar Vandana, Naseema Hayat Barlaskar, Abu Barkat Md Gulzar, Islamul Hoque Laskar, Diwakar Kumar, Prosenjit Paul, Piyush Pandey, Pranab Behari Mazumder

**Affiliations:** 1 Department of Biotechnology, Assam University, Silchar, Assam, India; 2 Department of Microbiology, Assam University, Silchar, Assam, India

**Keywords:** Bacteria, dysbiosis, immunoglobulin, Gut microbiome, short chain fatty acids, cancer

## Abstract

The human gut is rich in microbes. Therefore, it is of interest to document data to link known human diseases with the gut microbiota. Various factors like hormones, metabolites and dietary habitats are responsible for shaping the microbiota of the gut. Imbalance in the gut microbiota is responsible for the pathogenesis of various disease types including rheumatoid arthritis, different types of cancer, diabetes mellitus, obesity, and cardiovascular disease. We report a review of known data for the correction of dysbiosis (imbalance in microbe population) towards improved human health.

## Background

Bacteria emerged 3.8 billion ago while archaea, protists, fungi, and bacteria were separated as non-symbiotic single cells although some are host associated [1,2]. Thus, the host and its emerged microbial communities stretch the phylogenetic tree [3,4]. The microbiome was coined as a result of the collection of genome content of microbial cells or the microbial metagenome, however, the term microbiome and microbiota are recently used in exchange [1-5]. The microbiomes are co-evolved along with the host that shaped the appearance in our ancestral lineages. Natural selection and mutation have helped in evolutionary adjustment to environmental conditions [6-8].

Immune system together with microbionts possess a complex mechanism to prevent microbial colonization and destroy the invading microbes [9]. A new born baby has a paramount difference in gut microbiota as compared to a one-month-old baby and a six-month-old baby [10]. In human, the gut is the habitat for a huge and active bacterial community [11]. Different bacterial species are evolved and about 300-1000 different species are the natural habitat in the human gut [12-14]. A few microbial cells in the gut are 10 times higher in number than the eukaryotic cells. The stomach and the small intestine, both contain less microbial species in the epithelial because of the composition of the lumen that secretes acids, bile, and pancreatic secretions, which kills many microorganisms and causes stable bacterial colonization in the lumen [15,16]. The large intestine, in contrast, contains a complex set of microbial community with a large accumulation of living bacteria approximately 1011 or 1012 cells/g concentration in the lumen of the intestine [17]. Host homeostasis is affected by many bacteria living inside the colonic lumen; some are potential pathogens which can cause infection and sepsis [18]. An interaction between the host and the microbial cells can also provide important health benefits to the human host [19,20]. The host and the microorganism together referred to as superorganisms [11]. The microbial habitat comprises of the oral cavity, respiratory tract, genital area, skin surface, and gastrointestinal system [21].The diverse and composite microbiota serves as a functional growth of the host genome, it is estimated to possess 50 to 100 fold genes, these extra genes contributes to various function [22]. The microbiota has a consequential and certain metabolic, trophic and protective role as obtained from the evidence of the effect of gut microbiota on host physiology [23]. The important function of metabolism is fermentation of the undigested dietary fat or protein residue, and as well as the fermentation of the mucus which is endogenously produced by the epithelial gut [11]. It also produces toxic substances like ammonia, nitrites, a nitrogen compound, amines, phenols, thiols. Fermentation is high in the right colon and caecum with high production of acid (pH-5-6), SCFAs (Short-chain fatty acids) and growth of bacteria [22,24]. Vitamin, calcium, magnesium, iron synthesis also takes place in the colon by the colonic microorganism [25]. SCFAs for example acetate, propionate, and butyrate helps in absorption of ions and regulates homeostasis [26,27]. SCFAs regulate the function of microglia, the tissue macrophage of the central nervous system (CNS) [28]. An inflammatory response is suppressed by T regulatory (Treg) cells which includes host tolerance in non-host cells, making gut microbiota to build niches in the host without being killed by host immunity under normal conditions [29, 30]. All SCFAs stimulate cell proliferation and differentiation of epithelial cell in the intestine. It also prevents ulcerative colitis and colonic carcinogenesis to some extent [31,32]. Host-microbial interaction plays a vital role in the mucus layer in developing the immune system [33].The interaction between the gut and lymphoid tissue leads to the development of complex mucosal and systemic immunoregulatory tissue. Surface diversity helps the organism to skip immune surveillance and retain an ecological niche in the intestinal tract [34]. The immune response of the host to microbes depends on innate and adaptive constituent such as immunoglobulin IgA secretion [35]. The innate immunity discriminates pathogenic bacteria from commensal bacteria and kills the pathogenic ones with the use of receptors, like Toll-like receptors (TLR), as well as elicits different types of cytokines which have signal transducing pathway and promote the change in the phenotype of lymphocytes [36].

**Figure 1:**
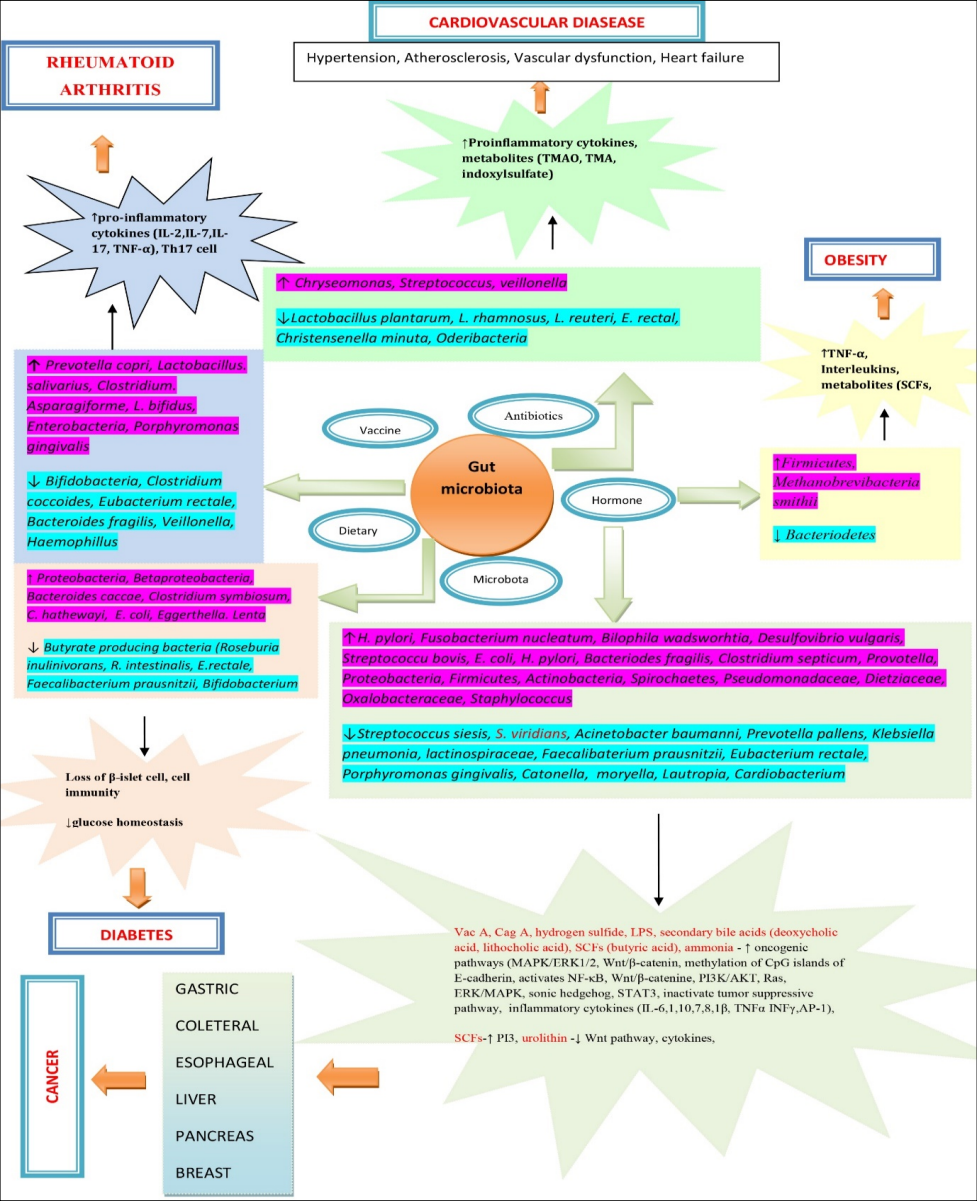
Gut microbiota and its impact on the cardiovascular system, cancer, obesity, diabetes and rheumatiod arthrritis.

Microbiota in the gut also provides colonization resistance from opportunistic bacteria. The equilibrium in the gut with species resident provides stability in the gut microbial population under normal conditions [37,38]. However, the use of antibiotics can disrupt the balance and causes an overgrowth of the pathogenic microorganism or opportunistic population [39]. When there is a gut microbiome perturbation, dominant non-pathogenic microbiota decreases in number and reduces the resistant of colonization capacity, which in turn allows opportunistic pathogens to colonize the niches and leads to infection [29]. Due to intrinsic complexity and multiplicity of the human microbiota, experiments are needed to investigate the cause and correlation linkage between microbiota imbalance and human disease [40,41]. However, the research on human gut microbiota is still in its preliminary stage. There is an interest to establish the relationship between human diseases and gut microbiota. Therefore, it is of interest to discuss distinct types of diseases that link to gut microbiota using known data in the literature and as illustrated in (Figure 1).

## Rheumatoid Arthritis:

Rheumatoid Arthritis (RA), an autoimmune ailment produces autoantibody and causes bone destruction in the multiple joints. RA is caused by various environmental factors such as microbiota, smoking, and hormones [42]. More than 100 hereditary susceptibility loci are associated with the development of RA. Previous reports suggest that the mucosal site is the origin of RA [43]. The mucosal site comprises of the oral cavity and the gut [44]. Several studies have revealed that the intestinal microbiota composition is altered during infection [45,46]. Vaahtovuo et al. investigated in patients with early RA utilizing a method like DNA staining and 16S rRNA hybridization, and found that the class Bifidobacterium, Clostridium coccoides and Eubacterium rectale, the subgroups Bacteroides fragilis are lower in RA patient [47]. In Japan, it was also confirmed that 33% of patients with RA had expanded Prevotella copri in the gut [46]. In China, the patients having RA had increased Lactobacillus salivarius presence in the gut microbiota, saliva, mouth, and tooth [48]. Based on the metagenomic shotgun sequences, it was found that Clostridium asparagiforme is abundant in the gut [48]. A periodontal bacterium, Porphyromonas gingivalis, major pathogenic bacteria, cause gum disease, is linked with the cause of RA, showed the presence of peptidyl arginine deiminase and might be related to ACPA [49]. Patients having RA have shown an increased number of P. copri an obligate anaerobe [46]. P.copri induces Th17 cell-dependent joint inflammation (arthritis) in mice [50]. Animal model studies revealed that by mono-colonization it is sufficient to develop arthritis of germ-free SKG (Streptokinase G) mice with P. copri [42]. Under germfree condition, the mice showed no development of arthritis but mono-colonization mice with Lactobacillus bifidus/P. copri cause arthritis by activation of Tol-Like Receptor 4 and 2 (TLR-4 and TLR-2) [51]. K/BXN transgenic mice showed inflammation (Th17 cell-dependent) in the spleen and small intestine by increasing Th17 cells. The auto-antibodies against Glucose-6-phosphate isomerase were reduced when the mice cared under GF condition, but by mono-colonization with SFB (segmented filamentous bacteria) developed Th17 cell-dependent arthritis [52]. Scher et al. investigated that P. copri abundance in the intestine is due to the nonattendance of human leukocyte antigen (HLA) DRB1 (DR Beta1) [53]. Intestinal microbiota-humanized mice are produced and the severity of the RA was analysed [46]. It was accounted for that SKG mice promote TH17 cell-dependent autoimmune arthritis, no symptoms of arthritis showed in the GF-SKG mice when served with a fungal constituent. It explained that for arthritis to develop, the microbial stimulus is required. But, when the faecal samples of RA patients are treated to GF-SKG mice, the mice developed into RA-SKG mice with a P. copri ruled microbiota. It was observed that the RA-SKG mice infused with low portion of zymosan (fungal element) developed severe arthritis [54,55]. Prevotella histicola in the human gut microbiota, a significant commensal bacteria, reduce the severity of arthritis in HLA-DQ8 mice [56]. When HLA-DQ8 mice treated with P. histicola, which is isolated from human duodenum, it showed a significant decrease of arthritis as a result of suppressing the serum level of various proinflammatory cytokines like IL-2, IL-7, and also the TNF-α (Tumor necrosis α). It showed decreased level of antigen-specific Th17 response with the increase of regulatory T cells in the digestive tract [56]. Maeda et al. reviewed the work of gut microbiota in human having RA and the mouse models having joint inflammation and observed that some species suppressed the development of arthritis [42]. Annalisa et al. found an HLA-DR-presented peptide (T cell epitope) from 27-kDa P. copri protein (Pc-p27) in the synovial tissue, peripheral blood mononuclear cells (PBMCs), and synovial fluid mononuclear cells of some RA patients by liquid chromatography-tandem mass spectrometry technique [57]. To study the response of Th1/Th17 in the peptides, stimulated PBMCs with the peptide are checked for the cytokines production with ELISA (enzyme-linked immunosorbent assay). It was discovered that interferon-γ (IFN-γ) level was about 42% in RA patients [58]. However, PBMCs from just a single patient having RA demonstrated showed increase in IL-17 [59].

In one study, it was observed that related taxa of Lactobacillaceae and lactobacillus were increasing in patients having RA and in the animal model of RA than in control, thus explaining that the bacteria as a inclining component of the RA disease [60,61]. L. casei and L. delbrueckii, administration was studied in experiment model to alleviate RA [62,63], and administration of L. rhamnosus GG and L. reuteri neglected to reduce the infection in patients having RA, explaining that various species act distinctively on RA [64-66]. Faecalibacterium, butyrate-producing bacteria helped in keeping up the health and integrity of gut epithelial and shows anti-inflammatory properties, thus they reduce the onset of the RA [67-69]. Gut microbiota like Blautia coccoides, Ruminococcus obeum and Bifidobacterium bifidum gut microbiota of systemic lupus erythematosus patients improved by immunosuppressive regulatory T cells (Tregs) production [70]. Methotrexate (MTX) considered as the first treatment strategy for immunosuppressive RA patients, but if the response is insufficient TNF-α inhibitors and other biotechnological treatment should be given [61, 71]. MTX can modify the microbiota organization, and reverses the disease-related dysbiosis [48]. Etanercept (ETN) treatment can partially restore the beneficial microbiota by modification. Anti-tumour necrosis factor-alpha (TNF-α)-ETN, change the beneficial microbiota presented in the gut [72,73]. Cyanobacteria and related phyla Nostocophycideae and Nostocales are enriched with ETN gathering and produce secondary metabolites which have anti-inflammatory and immunosuppressant activities help RA patients [74,75]. Anti TNF-α therapy has an advantageous impact on the microbiota synthesis [76].Later on, more studies and investigation are expected to understand whether P. copri serve arthritis contrasted with other intestine microscopic organisms [60].

## Cancer:

In human, gut microbes play an important role in cancer progression and also in prevention [77]. Gut microbiota regulates several different types of cancer [78]. When the undigested dietary components reach to the large intestine, mucin gets fermented by the anaerobic microbes found in the gut, which eventually produces a wide range of metabolites [79]. Among these, some of them are system- protective and some are destructive metabolites. The protective metabolites include SCFAs, glucose, succinate, propionate, D-lactose, L-lactose, etc. [80]. Whereas, bacterial subsets like Firmicutes and Bacteroides sp. ferment aromatic amino acids and generate some important bioactive products like phenols, indoles, p- cresol and phenyl acetic acid. Nitrogenous compounds like N-nitroso compounds (NOCs) shows the carcinogenic effect which has the potential to promote cancer through mutation by alkylation of DNA [80]. Pathogens like Shigella Flexner, Typhimurium, Helicobacter pylori, Streptococcus pneumoniae, Bacteroides fragilis and Salmonella enterica sub sp. enterica serovar produces some polyamines like spermidine, spermine polyamines, and putrescine in the gut which in due course causes cancer, oxidative stress, etc. [81]. A diverse group of gut microbes plays a significant role in certain cancer types.

Gastric cancer is amongst the five largest cancer types with more than a million incidences as in 2018 [82]. Gastric microbiota has both beneficial as well as harmful effects on human health. Some of them help in the digestion of complex food material, drug absorption and provide a safeguard against other pathogens [83]. A few of the parasitic microbes cause infection and other health issues. For a high time, the gut was considered to be bacteria-free because of its very low pH environment before the discovery of Helicobacter pylori in 1982 and was classified as a type I carcinogen [84]. Corpus-predominant gastritis is caused by H. pylori infection that leads to a condition known as hypochlorhydria. During hypochlorhydria, lower production of gastric acid occurs, which in turn may cause atrophic gastritis, an early stage of gastric cancer [84]. Production of low gastric acid makes an ideal environment for some nitrogen reducing bacteria that convert nitrates or nitrites from the saliva and dietary substrates further produce carcinogenic N-nitroso compound which causes damage to DNA and methylation of epithelial cells promotes carcinogenesis of the stomach. High dietary salt intake promotes gastric carcinogenesis [85]. Epidemiological reports showed that a high intake of salt is related to the incidence of H. pylori infection. The production of cagA is high with the intake of dietary salt and also elevates the inflammatory cytokines (IL-6, TNF-α, and IL-1) production [85]. The two fundamental components which induce gastric cancer are Cytotoxin-Associated Gene A (CagA) and Vacuolating Cytotoxin A (VacA) [77]. VacA promotes vacuolation of cells and autophagy, activates oncogenic pathways like mitogen-activated protein kinase (MAPK) and extracellular signal-regulated kinases (ERK1/2), and inhibits phosphatidyl-inositol 3-kinase (PI3K) pathways to alter cell division, cell proliferation, and cell death [86,87]. It is found to see that the person who is infected by the H. Pylori subdued the levels of ghrelin, a hormone that is found in the stomach that signals hunger and appetite [88, [89]. Vac A promotes methylation of CpG island of E-cadherin and activates Wnt/β-catenin pathway. Cag A upraise the accumulation of cytokines responsible for the inflammation like interferon-γ, IL-6, IL1, IL10, IL8, IL1β, TNFα and IL-7[87][90]. It activates NF-κB, Wnt/β-catenin, PI3K/Akt, Ras, ERK/MAPK, sonic hedgehog and STAT3 is disorganized with the infection of H. Pylori CagA+ strains [89] [88]. It also inactivate the tumor suppressor pathways by mutation of P53 [90]. Although all H. pylori strains can lead to gastritis but only the strains that bears Cag pathogenicity Island (cag PAI) carrying the cag gene has the ability to cause gastric cancer. Moreover, apart from H. Pylori, reduction of different other bacteria like Streptococcus sinensis, Acinetobacter baumannii, Prevotella pallens, Klebsiella pneumonia, Lactobacillus colihominis, and Lachnospiraceae are also responsible for gastric cancer in the human [91][92][93]. Protein components derived from pathogenic Helicobacter species like phospholipase C-gamma 2 proteins of outer membrane, nickel-binding proteins, and BAK protein, allows microbes to colonise the mucosal layer of gastrointestinal tract causing gastritis which induces tumorigenesis in the stomach [94].

Among all body parts, cecum and colon are the most populated microbial habitat in the body [80]. In a recent review on colon cancer reported that by producing genotoxic compound (hydrogen sulphide), Sulphide bacteria such as Fusobacterium, Bilophila wadsworthia and Desulfovibrio causes CRC by damaging DNA through chromosomal mutability [95,96]. Cell line studies demonstrated that genome mutability leads to high-frequency mutations over 80% in CRCs. This study suggests the role of hydrogen sulphide producing bacteria in developing CRC by the multistep carcinogenic process [96]. Sulphate-reducing bacteria, Desulfovibrio vulgaris, increases in number with the consumption of high-fat diets which produces secondary bile acids like deoxycholic acid and lithocholic acid by converting primary bile acids, leading to tumorigenic [97]. The sulphate-reducing bacteria produce LPS, the cell expressing subunits of LPS receptor, stimulates cellular immune response through TLR2, activating proinflammatory cytokine signalling which causes tumorigenesis [98-100]. Short-chain fatty acid like butyric acid (BA) produce by fermentation of fibres have resulted in anti-tumorigenic by colonic bacteria like Eubacterium rectale and Faecalibacterium prausnitzii [101,102]. Immune response like receptor of SCFAs, GPR109a expressed by a cell, stimulates the ligands of BA which helps to maintain cell proliferation, inhibits inflammatory cytokines by controlling the inflammation process [103,104]. It also expresses the anti-carcinogenic effect by inhibiting DNA-methylation which is mediated by GPR109a silencing through IFNc [104,105]. Metabolites of intestinal microbiota like urolithin A with ellagic acid have shown to suppress Wnt signalling pathway [106,107]. Inflammatory mediators like TNFα, IL1β, IL6 and many other cytokines produced by chronic inflammation activates NF-κB which causes colon carcinoma [108]. Fusobacterium produces a virulence factor in the cell surface called fusobacterium adhesion A(FadA) which is observed in patients having CRC. On the endothelium, FadA binds with E-cadherin and regulates the E-cadherin pathway or the b-catenin pathway and increase transcription factors, oncogenes and inflammatory genes [109] [110,111][112,113]. It also influences epithelial cell growth and cell proliferation. Studies show that Streptococcus bovis (S. bovis), Escherichia. Coli (E. coli), Fusobacterium nucleatum (F. nucleatum), Helicobacter pylori (H. pylori), Bacteroides fragilis (B. fragilis), Clostridium septicum (C. septicum) were found predominantly in CRC patients when compared to controls [114, 115]. Fusobacterium nucleatum, a putative bacterium is found abundantly in the cancer tissue which promotes the tumorigenesis of intestine through binding to cancer cells and regulating immune system by its Fap2 proteins [116][117][118]. The putative bacterium also regulates β-catenin cancer pathway, microRNA-21 expression, TLR 4 to induce autophagic pathway, related microRNAs, and, Fab2 can bind to T cell immunoglobulin and immune-receptor TIGIT (tyrosine-based inhibition motif domain) which is an inhibitory receptor present on the NK (natural killer) cells and lymphocytes, resulting in inhibition and causes suppression of immune response [80]. Downregulation of the immune system allows immune inflammatory response promoting into potential CRC [80].

Oesophageal cancer ranks sixth among reasons for deaths related to cancer and eighth in most regularly diagnosed cancer [119]. It was observed that the most common microorganism in the normal oesophagus is Streptococcus viridians with the occurrence rate of 95-98% [120][121]. A study conducted on the normal oesophagus by using 16S rDNA sequencing recognized 95 species in six phyla containing Firmicutes (eg Streptococcus), Fusobacteria (eg Fusobacterium), Actinobacteria (eg Rothia), Proteobacteria (eg Haemophilus), Bacteroides (eg Prevotella), and TM7 [122,123]. This malignant disease is been subdivided into two groups namely esophageal squamous cell carcinoma (ESCC) and adenocarcinoma (EAC) [77]. It was found that gram-positive (+ve) bacteria like Firmicutes and Streptococcus are present mainly in the normal esophagus whereas the gram-negative (-ve) bacteria like Spirochaetes, Bacteroidetes, Fusobacteria are found associated in Barrett's oesophagus and esophagitis [124]. Lipopolysaccharide (LPS) produced by the gram-negative bacteria regulates the innate immune system directly or indirectly by a different mechanism. It is regulated by the innate immune response, which ultimately results in NF-κB activation, stimulating the pro-inflammatory mediators like IL-6, IL-1β, IL-8 and TNF-g [125][126]. It also increases the level of nitric oxide (NO) and inducible nitric oxide synthase (iNOS) in the lower oesophageal sphincter [127]. Gastroesophageal reflux disease (GERD) leads to Barrett's esophagitis, one of the significant factors for EAC [128]. In a study, it is reported that 0.5-1% of GERD positives cases with Barrett's oesophagus have chances to develop adenocarcinoma. Population-based meta-analysis study indicated that there is a fivefold enhanced risk of EAC in the population with weekly GERD symptoms [129]. Interestingly, on the other hand, being the class-1 carcinogen H.pylori is found in reducing the risk rate of oesophageal adenocarcinoma and oesophageal squamous cell carcinoma. H.pylori suppresses EAC by decreasing the level of pH as they prevent the parietal cells to secrete HCl [130]. Experimental investigation has been conducted between the microbiome of oesophageal adenocarcinoma and the normal epithelium, it was observed that the composition differs in both the groups with the upregulation of E. coli and TLR 1-3, 6, 7 and 9 in the oesophageal adenocarcinoma [131]. In oesophageal squamous cell carcinoma (ESCC), the microbiomes are less characterized than in the oesophageal adenocarcinoma (EAC) [131]. Porphyromonas gingivalis is present in patients having oesophageal squamous cell carcinoma and serves as a biomarker for clinical purpose [132]. It was also observed that the genera Catonella, Moryella, Lautropia, Peptococcus, Bulleidia Corynebacterium, and Cardiobacterium is less in number in the saliva of the patients with oesophageal squamous cell carcinoma [133].

In the liver, Cholangiocarcinoma (CCA) and Hepatocellular Carcinoma (HCC) are two main histological types of cancer. HCC is mainly caused by aflatoxin B1 (AFB1), alcoholic liver disease (ALD), hepatitis B and C virus, and non- alcoholic fatty liver diseases (NAFLD) [134-136]. HCC patients have a number of E.coli in comparison to the healthy controls whereas Pseudomonadaceae, Oxalobacteraceae and Dietziaceae are found more in CCA patients [137]. H. pylori produce VacA and CagA in the HCC positive liver which promotes the growth of liver cancer [138,139]. It also produces LPS that directly promotes the growth of liver cancer by elevating the level of TGFβ1 and IL8 [140]. H. hepaticus, member of the family Helicobacteraceae, is also a causative organism for liver cancer [141]. They activate the NF- κ B, oxidative stress, Wnt signalling pathway, and hepatocyte turnover thus resulting in HCC [141]. Bacterial metabolites like LPS (lipopolysaccharide) is been recognized by the TLR4 and activates Kupffer cells by LPS induced IL6 and TNFβ [142]. This LPS-TLR4 pathway regulates HCC whereas eviction of LPS of TLR4 inactivates and decreases the rate of HCC development [143]. Primary bile acids like cholic acid and chenodeoxycholic acid produced by the liver causing DNA damage by producing reactive oxygen species (ROS), resulting in the development of liver cancer [144]. Commensal bacteria of gut modulates antitumor immunity by regulating the metabolism of primary bile acids to secondary bile acids which are circulated back to the liver by the enterohepatic circulation [145]. Investigating by altering the gut commensal bacteria induce an antitumor effect in the liver, it increases hepatic CXCR6^+^ NKT (natural killer) cells which leads to inhibition of growth of tumour in the liver [145].

Pancreatic Ductal Adino Carcinoma (PDAC) is most commonly found and is the fatal pancreatic cancer worldwide [146]. It is observed that H. pylori infection is a risk factor for PDAC, acute autoimmune and chronic pancreatitis [147-149] Microbial metabolites like LPS and ammonia are secreted from H. pylori as well as inflammatory cytokines like IL-7 which cause inflammation and damage to the pancreas; it also stimulates NF-κB,STAT3 and AP-1 resulting in upregulation of the cellular process, anti-apoptotic expression and pro-proliferative proteins like MCL-1, cyclin-D1, Bcl-xL, and c-myc [150] [151-153]. There are different types of microbes found in the gut system which cause the inflammatory reaction and immune response that is responsible for pancreatic cancer [154]. TLRs are expressed on the immune cell and recognizes microbe-associated molecular patterns (MAMPs) and damage-associated molecular patterns (DAMPs) that stimulate the MAPK signalling and NF-κB pathways which causes pancreatitis and finally pancreatic cancer [155,156]. Fusobacterium is present in the pancreatic cancer tissue and it is marked as a biomarker for clinical diagnosis of pancreatic cancer [157].

Mutation in the BRAC1 and BRAC2 gene leads to breast cancer [158]. Dysbiosis of the gut microbiome leads to breast cancer. Metabolites are secreted by the gut like reactivated oestrogens, modulating the estrogen serum level, SCFC, amino acid, secondary bile acid regulates breast cancer [159]. Studies show that the microbes of a healthy woman are different from the diseased one [159]. Dysbiosis characterizes several diseases which include breast cancer [160][161]. In breast cancer, dysbiosis also occurs in the breast's own microbiome [159]. When tissue from breast tumour and normal adjacent tissue are taken it was observed that the number and size of bacteria measured by the copy number of 16srDNA are less in the tissue of breast cancer [162]. It showed changes in the microbiome, it has abundant phyla of Proteobacteria, Actinobacteria, Firmicutes, and Bacteroidetes in the breast tissue. It was also observed that Methylobacterium radiotolerans increases in tumour tissue [163]. In Canada, it was observed to have a diverse type of population in the patient suffering from breast cancer. It showed richness in the taxa: 11.4% of Bacillus, 6.5% of Staphylococcus, 5.0% of Prevotella, 5.8% of Propionibacterium, 5.7% of Comamonadaceae, 5.0% Gammaproteobacteria, 6.5% of Pseudomonas sp [164]. It was also detected a higher number of E. coli in women having cancer in comparison to the healthy controls [164]. Nipple aspirate fluid (NAF) from women with breast cancer and healthy women was compared; it resulted in a significantly different composition of microbiomes. It showed an increase in beta-glucuronidase level and genus Alistipes abundance in NAF patients than from healthy controls [165]. It was estimated that breast cancer tissue is enriched with Atopobium, Hydrogenophaga, Fusobacterium, Lactobacillus Bacillus, Gluconacetobacter, Enterobacteriaceae, Faecalibacterium, Staphylococcus, Clostridiaceae, and Ruminococcaceae [166-168].

## Obesity:

Obesity is characterised by accumulation of visceral fats [169], which is caused by many factors like diabetes, cardio-disease, hypertension, dyslipidemia, fatty liver and cancer, it is also caused by inflammatory molecules like TNF-α (Tumor Necrosis Factor Alfa) and Interleukins, which is observed to cause obesity [170][171]. Recent statistical information from WHO (World Health Organisation) showed that 300 million people are obese and one billion people are overweight worldwide [172] Obesity has turned a worldwide issue and gut microbiota assumes an imperative job in the organization and the advancement of obesity [173-175]. The mechanism is mostly unclear. Nonetheless, it has been reviewed that gut microbiota causes obesity by affecting various variables like increase lipoprotein lipase action (LPL), host genome, lipogenesis, inflammation, expanded calories, intestinal penetrability, consumption of food and use of energy [173]. A recent report explained that specific bacteria are involved in the uptake of nutrient and homeostasis of energy. Bacteria producing LPS (lipopolysaccharide) from the gut, trigger factor and link inflammation to fat-rich eating routine causes obesity [176,177]. Recent studies have shown gut microbiota role in fat storage and energy homeostasis [169]. High creation of SCFA (short-chain fatty acid) in the gut by Methanobrevibacter smithii causes obesity [178] [179]. In particular, the investigation was done on mouse model showed enhanced SCFA production by Methanobrevibacter smithii, the strain in the human gut directly induces obesity development [179]. Obesity is associated with gut microbiota composition as it was detected recently by metagenomics techniques like16S rRNA gene sequencing, RT-PCR, fluorescent in-situ hybridization [180][181]. Obesity is observed as an essential risk factor for inducing T2DM and sequentially T2DM is a treat factor inducing Alzheimer's disease [182][183]. It has been studied that the vascular effect of obesity on the cardio-system causes a major problem in causing Alzheimer's disease, researchers are still trying to understand the molecular mechanisms, their link with the disease [184]. Phylum Firmicutes is more abundant and Bacteriodetes is less abundant in obesity as summarised by studies on animal models [185,186]. Increase evidence has showed that the composition of bacterial microbiota is connected to obesity [187]. In mice which are obese have Bacteroidetes less in number and Firmicutes more in comparison to lean mice [188]. In animal model such as mice model of obese, it was observed that with change in leptin gene it have less types of Bacteroidetes and more of the types Firmicutes in comparison with lean-wild sort mice, with similar type of diet are fed [188]. Bacteroidetes increases with the loss of weight by consumption of calories through fat-reduced or carbohydrate-rich diet, explaining that Bacteroidetes are dynamic in calories consumption [185]. When the carbohydrates are undigested, changed over into SCFA by breakdown by microbiota of gut, higher production is identified with expanded obesity rate. SCFA are oxidised in the animal model by the host that provide more calories and promote more fat and gain more weights [189]. The human with obesity has a gene-coding enzymes that degrade indigestible polysaccharides in the diet, which are found in the gut microbiota, thus allow greater energy extraction from the consumption of given food. In obese participants, it is studied that the permeability of intestinal to LPS and alteration of bile acid signalling cause greater energy efficiency [190].

## Cardiovascular disease:

The gut microbiota has also appeared to be an integral regulator of cardiovascular diseases. Bacterial structural elements and microbial metabolites had increased in the circulating level due to dysbiosis that can aid in the growth of cardiovascular disease (CVD) [191]. CVD involves various disorders like as, coronary artery disease, stroke, hypertension, and even a heart failure, which evoke preclinical disturbances in the vasculature, which include arterial stiffness and endothelial dysfunction. There are two catabolic pathways of digestion of food in the human gut microbiota, saccharolytic or proteolytic [192]. In the saccharolytic pathway, sugars are broken down in the gut and are the major site for SCFA production. The proteolytic pathway is associated with protein fermentation which produces SCFA and also some metabolites like amines, indoles, ammonia, thiols, and phenols, which are toxic and accumulation lead to microbial uremic toxins [193].

Hypertension is one of the most common disruptions of the cardiovascular system, which affects over one billion people across the world [194]. Experimental evidence has shown that dysbiosis due to the altered gut microbiota can lead to the uplift of blood pressure. Honours et al. provided the first evidence associating the gut bacteria with hypertension. In a series of experiments; he revealed that antibiotic treatment in rats has attenuated steroid-induced hypertension [195]. In a comparison of the microbiota of normotensive (having normal blood pressure), Wistar-Kyoto rats having spontaneously hypertensive showed the reduced microbial richness, multiplicity, and evenness, with an increase in Firmicutes: Bacterioidetes ratio [196,197]. Interestingly, changes associated with bacteria that produce acetate and butyrate were found to be reduced that might be responsible for contributing to the higher blood pressure. Genus Oderibacteria producing butyrate are abundant in overweight and obese pregnant women who is having low blood pressure. Toral et al. suggested that the sympathetic nervous system and the intestines play a role in hypertension by characterizing enhance gut-hypothalamic signalling, dysbiosis, permeability, intestinal inflammation were characterized in a murine model [198]. SCFAs produced by the gut stimulate G-protein coupled receptors (GPR) pathways which regulate blood pressure and rennin secretion [147].

The atherosclerosis pathophysiology is distinguished by a basic disrupt in the endothelial lining, which leads to the accumulation of lipids and release of macrophages and other cells of the immune system to the arterial wall [200,201]. Atherosclerosis plaques consist of miscellaneous species of bacterial DNA, was revealed by the association of the microbiota with atherosclerosis [202,203]. Various bacterial phylotypes of the same individual were identified from the sample of oral and gut, revealed that this microbiota serves as an initial source of atherosclerosis. The patient suffering from Atherosclerosis, have been observed with a reduced level of Roseburia which produces pro-inflammatory peptidoglycans and it also decreased the production of carotenes which have anti-inflammatory properties [204].

One of the clinically pertinent expressions of vascular dysfunction is the endothelial dysfunction, which could be a powerful independent risk factor for the cause of future cardiovascular diseases and mortality [205,206]. Karbach et al. 2016 observed that GF mice colonization contributed to mild endothelial dysfunction and lead to an increase in expression of tbx21, known to encode a key transcription factor for IFN-γ [207]. Significantly, conventional and GF mice showed related vasoconstrictive and vasodilatory responses, which suggests that the endothelial function was impaired by the process of colonization. Early marker for atherosclerosis is signalled by endothelium-dependent vasorelaxation. Gut microbe-Sirtuin1-vascular microRNA leads to the development of endothelial dysfunction. Microbiomes regulate the vascular microRNA-204 (miR-204) expression, and it also regulates the endothelial function by attacking the Sirt 1 (Sirtuin1) lysine deacetylase, in aortas of germ-free mice, triggered by feeding high-fat diet [208]. In mouse aortas, antibiotics suppression of the gut microbiota decreases miRNA-204 but increases bioavailable vascular nitric oxide and Sirt 1 and enhances endothelium-dependent vasorelaxation. Impaired endothelium-dependent vasorelaxation is rescued by the miR-204 systemic antagonism and vascular inflammation is decreased by the induction by high-fat diet [209]. Elevated systemic congestion and lowered cardiac output may cause abdominal mucosal ischemia or oedema, which increased bacterial translocation and rise of circulating endotoxins that caused inflammation with heart failure in patients signified by the gut hypothesis [210][211]. In a study, patients with heart failure have lower abdominal blood flow which showed to have large serum concentrations of immunoglobulin A-anti lipopolysaccharide, correlated with expansion growth of bacteria [212]. Recently a study stated that a corresponding growth in the amount of fecal intestinal fungi and bacteria with the rise in intestinal permeability in chronic heart failure patients in comparison to healthy controls [213]. Animal model studies recently suggested that the trimethylamine-N-oxide (TMAO) pathway directly contributes to heart failure by adverse ventricular remodelling [214]. TMAO, derived from dietary nutrients includes choline and carnitine, an increase in the blood levels has a direct link with patients having CVDs [215]. Even after traditional risk factor adjustment, it was observed to have a high level of TMAO with increased risk of CVDs, suggested that the gut microbiota role in the development and growth of CVDs [216]. Additionally, optical coherence tomography assessed the association of the blood TMAO levels with coronary plaque vulnerability and cardiovascular acute coronary syndrome [217].

## Conclusion and Future Direction:

The relationship between the host metabolism and the human gut has been discussed using known data in literature in this review. It showed differences in the gut microbiota contribution among different host causing dysbiosis leading to various form of diseases in human. The application of various molecular technique including animal model studies helped to understand the composition present in gut microbiota linking with human diseases and health. It helped to understand dysbiosis (imbalance in microbe population) to restore the beneficial microbes for healthy living. Data on the composition of microbiota and its contribution will help link with potential diseases and its cause for effective combat and care. This is associated with the imbalance contributing to different types of diseases. It should be noted that probiotics are often safe. However, Issues such as antibiotic resistance gene and virulence gene transfer among the different groups should be accounted before application. Application of microbiota-based treatment, prognosis, diagnosis, and monitoring will exhibit great role in transforming the current treatment in disease management.
